# Sleep and biological parameters in professional burnout: A psychophysiological characterization

**DOI:** 10.1371/journal.pone.0190607

**Published:** 2018-01-31

**Authors:** Arnaud Metlaine, Fabien Sauvet, Danielle Gomez-Merino, Thierry Boucher, Maxime Elbaz, Jean Yves Delafosse, Damien Leger, Mounir Chennaoui

**Affiliations:** 1 Université Paris Descartes, Sorbonne Paris Cité, Hôtel Dieu, Paris, France; 2 APHP, Hôtel Dieu, Centre du sommeil et de la vigilance, Paris, France; 3 Unité Fatigue et Vigilance, Institut de recherche biomédicale des armées (IRBA), Brétigny sur Orge, France; 4 Bio Paris Ouest, Courbevoie, France; Charite Medical University Berlin, GERMANY

## Abstract

Professional burnout syndrome has been described in association with insomnia and metabolic, inflammatory and immune correlates. We investigated the interest of exploring biological parameters and sleep disturbances in relation to burnout symptoms among white-collar workers. Fifty-four participants with burnout were compared to 86 healthy control participants in terms of professional rank level, sleep, job strain (Karasek questionnaire), social support, anxiety and depression (HAD scale). Fasting concentrations of glycaemia, glycosylated hemoglobin (HbA1C), total-cholesterol, triglycerides, C-reactive protein (CRP), thyroid stimulating hormone (TSH), 25-hydroxyvitamin D (25[OH]D), and white blood cell (WBC) counts were assessed. Analysis of variance and a forward Stepwise Multiple Logistic Regression were made to identify predictive factors of burnout. Besides reporting more job strain (in particular job control p = 0.02), higher levels of anxiety (p<0.001), and sleep disorders related to insomnia (OR = 21.5, 95%CI = 8.8–52.3), participants with burnout presented higher levels of HbA1C, glycaemia, CRP, lower levels of 25(OH)D, higher number of leukocytes, neutrophils and monocytes (P<0.001 for all) and higher total-cholesterol (P = 0.01). In particular, when HbA1c is > 3.5%, the prevalence of burnout increases from 16.6% to 60.0% (OR = 4.3, 95%CI = 2.8–6.9). Strong significant positive correlation existed between HbA1C and the two dimensions (emotional exhaustion and depersonalization (r = 0.79 and r = 0.71, p<0.01)) of burnout. Models including job strain, job satisfaction, anxiety and insomnia did not predict burnout (p = 0.30 and p = 0.50). However, when HbA1C levels is included, the prediction of burnout became significant (P = 0.03). Our findings demonstrated the interest of sleep and biological parameters, in particular HbA1C levels, in the characterization of professional burnout.

## Introduction

The professional burnout is a psychological syndrome occurring in response to chronic job stress. The most widely used Maslach Burnout Inventory (MBI) measures burnout across the three dimensions, emotional exhaustion (EE), personal accomplishment (PA) and depersonalization (DP) [1,2]. It is an outcome of chronic depletion of the individual’s coping resources resulting from prolonged exposure to stress, particularly work-related stress [3]. Burnout in workplaces has been attributed to different factors, which can be classified as organizational and individual factors [3] and occurs in workers as a result of failure to cope with occupational stress [3]. Originally, the burnout symptoms were found to occur only in client-related occupations [4], but now include all types of work [5]. Most of the studies that estimate the prevalence of burnout focused on different occupational group primarily service—related professions with rates ranging from 25 to 60% [6–9]. With respect to stress measurement, the demand-control-support model of Karasek [10] is classically used and posits that job strain results from the joint effects of high job demand and low job control. This model of work stress has been widely used to examine adverse effects on health, such as type 2 diabetes and cardiovascular diseases, and mental illness [11]. There are now growing evidence supporting its negative implications for mental and physical health and recently accumulated evidence suggests that it may be considered a risk factor for severe injuries [1,3,12]. Thus, chronic job strain, has been found to predict cardiovascular disease [13], type 2 diabetes [13,14], and poor self-rated health [15]. High job strain increased the risk of mortality particularly in white-collar men [16]. Several epidemiological studies have implicated biological parameters such as level of glycosylated hemoglobin (HbA1C), which positively correlates with stress level regardless of the method of stress assessment: “job strain” with job demand-control model [17,18], or “job stress” evaluated by the effort-reward imbalance (ERI) model [[19].

Actually, burnout is not recognized as a disease and its definition remains uncertain, and there are no current standardized diagnostic criteria [20]. Some authors suggest that it is less a medical condition but more a phenomenon related to the match between a worker’s and his / her workplace’s expectations, and that this should be approached by management techniques rather than by treatment [21]. Fatigue, mood disorder, sleep problems and cognitive impairment seem to be the most common symptoms of burnout [22]. The fatigue is a dominant component in burnout but alternative or complementary factors could be disturbed sleep and mental disorders [23–25]. However, besides the mental health consequences of burnout, the negative physical health impact is important. In 2003, Grossi et al. [26], find burnout to be positively associated with HbA1C and TNF-α among women. The review of Melamed et al., (2006) [27] enlightened association between burnout and a subset of bio-clinical parameters (i.e. the metabolic syndrome, type 2 diabetes, systemic inflammation, impaired immunity functions) that may impact health more extensively than currently suggested. For them, allostatic load might be a mediating mechanism. However, the most recent systematic review on burnout, incorporating different biomarkers, has found no convincing relationship between biomarkers and burnout. For these authors, the “methodological inconsistencies” lead to the incomparability of studies [28].

We hypothesized that chronic exposure to job strain which leads to burnout has a specific biological signature, in particular metabolic and inflammatory responses. Our aim was to assess potential clinical and biological variables associated with burnout in a group of white collars workers using a cross-sectional multidimensional study. We focused on biological parameters, job strain and social support, and sleep and mental disorders. Symptomatic burned-out workers were compared to control healthy workers who were selected during the occupational medicine survey in a French financial company.

## Materials and methods

### Study design

We constructed a case-control study from a French working population. All subjects who were included were regular contract employees from a large financial French company. They were included during a three-year period study between September 2012 to September 2015. The analysis included white collar workers with symptomatic burnout (cases) and examined differences in clinical, professional and biological outcomes with non-burnout workers (healthy controls) from the same company. Study purposes and procedures were explained to the employees, and written informed consent was obtained from them prior the study. The procedures were in accordance with the Declaration of Helsinki and the study was approved by the Ethical Committee of the Hôtel Dieu Hospital, Paris, France.

### Subjects

Cases were defined as subjects with no history of physical or mental disorder who consult as a matter of urgency for a psychological complaint or distress spontaneously related to work stress environment at the company’s occupational health center on site. All questionnaires, clinical interviews and examinations were conducted by the occupational physician and secondly addressed to a psychiatrist if necessary.

Subjects with a mood disorder, alexithymia, and other mental conditions such as anxiety disorders (generalized anxiety disorder, panic disorder, specific phobia, post-traumatic stress disorder…) that could overlap with burnout were excluded. During the period study, 9 subjects were addressed to the psychiatrist and were excluded because of a chronic severe psychiatric disorder (3 with major depressive disorders, 2 with personality disorders, 2 with a bipolar disorder, 1 panic disorder and 1 PTSD). Second, we limited enrollment to subjects without a history or previous diagnosis of metabolic and endocrinological problem, coronary heart disease (CHD), inflammatory diseases, allergies. Two subjects were excluded: 1 with hyperthyroidism and 1 with a type 2 diabetes.

After excluding other diagnosis and assessing the severity of burnout using the Maslach Burnout Inventory (MBI) questionnaire [2], each subject was included in the study.

Healthy controls came from the same company and were selected at random from other regular contract employees during the routine occupational health examination performed at the same day or week during which each case was included. They were defined as subjects who do not experience the outcome (i.e. burnout syndrome) during the period study. We matched every person with burnout as closely as possible by age, sex, BMI, and income category with healthy controls.

After an overnight fast, all participants were invited to a morning lab visit. This included the assessment of socio-demographic and work characteristics using self-report questionnaires; including age, gender, weight, body mass index (BMI), occupational rank, and subjective quality of life using a visual analogue scale (VAS).

During the study period, 54 burnout subjects have been included (30 men and 24 women) and matched with 86 healthy controls (41 men and 45 women).

### Data collection

#### Burnout

The severity of burnout symptoms was assessed with the widely used Maslach Burnout Inventory (MBI). It is a questionnaire consisting of 22 items, distributed in three dimensions [2]: 9 items for Emotional Exhaustion (EE) (a drained, depleted feeling arising because of excessive psychological and emotional demands), 5 items for depersonalization (DP) (tendency to view others in an excessively detached, impersonal manner), and 8 items for Professional accomplishment (PA) (a sense of competence and accomplishment).

For the categorical definition of burnout, the cut-offs were chosen according to the scoring guidelines by MindGarden (Menlo Park [CA], USA). Burnout was identified at a high level (as opposed to low) based on the following scores: EE > 27, DP > 13 and PA < 30. A diagnosis of burnout (yes/no) was assigned if respondents presented high levels in at least two dimensions (either EE or/ and DP, associated or not with a low PA).

In this study, in order to analyze the internal consistency of the three domains of the Maslach Burnout Inventory (emotional distress, depersonalization and low professional fulfillment), the alpha Cronbach coefficient was respectively 0.86 for Emotional Exhaustion, 0.71 for depersonalization and 0.70 for personal achievement.

#### Psychosocial working conditions

They were evaluated from job strain levels which delineate job control and job demand scores from the validated French version of the Karasek job content questionnaire [11] completed during the occupational medical visit. Participants were asked to answer questions about the psychosocial aspects of their job. The response categories were a Likert-scale ranging from 1 (strongly disagree) to 4 (strongly agree). For each participant, mean response scores were calculated for job-demand items (i.e, questions about whether the participant had to work very hard, had excessive amounts of work, conflicting demands, or insufficient time) and job-control items (i.e., questions about decision freedom and learning new things at work). Scores on the job demands scale range from 12 to 48, with higher scores (> 24) representing higher demands. Scores on the job control scale range from 24 to 96, with higher scores representing higher control (> 72). Cut-off for each dimension was dichotomized to the median to define high and low score, from our previous study with a largest sample of the working population to which every included subject belongs [23]. In our study, the following Cronbach’s alpha coefficients were: job-demand (0.78), job control (0.76).

#### Sleep disturbances

Insomnia and other sleep disorders were assessed using a self-administered questionnaire, derived from the ‘Sleep Disorders Questionnaire—French version’ (SDQFV). The SDQFV is a 42-items questionnaire based on the “Stanford Sleep Questionnaire and Assessment of Wakefulness”. The French version has been validated in epidemiological studies [23,29].

In our study, we used part of the questionnaire that focused on sleep habits and insomnia. To assess insomnia symptoms, we add questions based on new reference documents: the “International Classification of Sleep Disorders” (ICSD-3) and the “Diagnostic and Statistical Manual of Mental Disorders”, 5^th^ revision (DSM-5). Clinical reappraisal was carried out with all subjects by an occupational health and sleep medicine specialist. The clinical interviews were carried out to establish a diagnosis of insomnia with DSM-5. Insomnia was assessed using the following questions: “Do you have difficulty falling asleep at night?” (Difficulty in initiating sleep), “Do you wake up during the night after you have gone to sleep and have difficulty getting back to sleep?” (Difficulty in maintaining sleep), and “Do you wake up too early in the morning and have difficulty getting back to sleep?” (Early morning awakening). Each positive response was considered as “trouble” if occurring at least during “three nights per week”.

The DSM-5 criteria for insomnia include difficulty of initiating sleep, difficulty of maintaining sleep, and early morning awakening, for a period of ≥ 1 month. In addition, it is a prerequisite that sleep disturbance significantly impairs daily function. Severity of insomnia was divided in two groups: moderate insomnia (one trouble) and severe insomnia (at least two troubles). Non-restorative sleep disorder was assessed separately using the question “How frequently are you bothered because your sleep is not refreshing, you don’t feel rested even if the duration of your sleep is normal.” Non-restorative sleep was considered present when it was reported to occur 3 to 4 times a week or more and lasted for at least 1 month. Excessive sleepiness was assessed using the “Epworth Sleepiness Scale” (ESS) [30]. Based on the total score, patients were classified in two subgroups of sleepiness: no sleepiness = 0–10, excessive sleepiness > 10.

#### Mental health (anxiety and depression)

Symptom levels of depression and anxiety were assessed by the Hospital Anxiety and Depression Scale (HADS) [31] consisting of seven items for each dimension. Answers are coded on a 4-point Likert scale (0 = not at all, 3 = mostly), giving rise to a total depression and anxiety score ranging between 0 and 21 points. A > 10 score is considered as certainly symptomatic.

#### Biochemical parameters

Venous blood samples were collected in EDTA tubes between 8 and 10 a.m. in seated subjects who had fasted overnight. White blood cell (WBC) counts were determined with automated blood cell counters. Serum total-cholesterol, HDL-cholesterol, triglycerides, C-reactive protein (CRP) and glucose were measured using the automatic analyzer COBAS (Roche Diagnostics, Switzerland), all reagents were prepared according to the manufacturer’s instructions. LDL-cholesterol was calculated using the Friedewald formula. 25[OH]D3 was measured in one batch using the Roche Cobas E601 analyzer, according to the manufacturer’s instructions (vitamin D3 [25-OH] assay with polyclonal antibody; Roche Diagnostics, Burgess Hill, UK). Thyroid stimulating hormone (TSH) was measured by electrochemiluminescence immunoassay (Roche Cobas E 601, module immunology analyzer, Roche Diagnostics, Burgess Hill, UK). The glycosylated hemoglobin (HbA1C) test was performed using reagents, calibrators and control materials from Bio-Rad D 100 ion-exchange high-performance liquid chromatography (HPLC) (Bio-Rad Laboratories, Inc. USA) according to the manufacturer’s instructions.

### Data management and statistical methods

A database was developed using the software Sphinx Plus^2^ (V 5.1, Le Sphinx development, Chavanod, France) and statistics tests were made using R studio (Version 0.99.175–2009–2014 RStudio, Inc.) and significance (α risk) was fixed at p<0.05. Continuous variables were presented as mean ± standard deviation (SD) and means were compared (Burnout vs. Control) using a 2-tailed t test. Dichotomous variables were presented as occurrence and percentage (n (%)). A χ^2^ (or Fisher exact test) was used to test the relationship between the variable and Burnout. When significant, odd ratio and his 95% confidence interval (OR [95% CI]) was calculated. Dependences between quantitative variables was checked using a Pearson correlation test (r ≥ 0.6 and p<0.05). Dependence between quantitative variable and burnout was test using a one-way analysis of variance (ANOVA). A multiple logistic regression was made to identify factors with outcome and estimate the probability for burnout diagnostic. Variables were entered as independent variables and presence of burnout was entered as the dependent variable. A value of p < 0.05 for the Wald criterion was considered to denote regression coefficients significantly different from zero. The results are shown as odds ratio (OR) with 95% confidence intervals (95% CI) for ORs. The fit of the models was judged by the Likelihood Ratio Test Statistic.

A Forward Stepwise Multiple Logistic Regression was made to identify predictive factors (independent variables) of burnout (dependent variables). We included in the models the independent factors significantly associated with burnout, in the model 1: Job satisfaction, HADS anxiety, Job control and Job demand, in the model 2: + insomnia, in the model 3: + HbA1C and in the model 4 + insomnia and HbA1C in order to evaluated respective influence of HbA1C and insomnia in the diagnosis of burnout. A value of p < 0.05 for the Wald criterion was considered to denote regression coefficients significantly different from zero. The results are shown as odds ratio (OR) with 95% confidence intervals (95% CI) for ORs. The fit of the models was judged by the Likelihood Ratio Test Statistic.

## Results

We compared 54 men and women aged 37.1 ± 7.4 y with symptomatic burnout to 86 healthy individuals matched for age, sex, BMI and professional rank. As expected, subjects with burnout scored higher on the two-related burnout dimensions, EE and DP, while lower on the PA score, compared to participants without burnout (Table 1).

**Table 1:** Sociodemographic and psychological characteristics of burnout and non-burnout (control) subjects.

	Control	Burnout	Anova p-value	χ^2^, OR (95% CI)
Number, n	86	54		
Women, n (%)	45 (52.2%)	24 (44.4%)		NS
Age, yr	30.8 ± 7.1	31.7 ± 7.4	0.51	
Weight, Kg	68.3 ± 13.4	67.0 ± 13.1	0.55	
BMI, kg/m^2^	22.7 ± 3.2	22.4 ± 3.2	0.57	
Rank /Level, n (%)				
Assistant	6 (7.0%)	4 (7.4%)		NS
Associate	2 (2.3%)	2 (3.7%)		NS
Director	3 (3.4%)	2 (3.7%)		NS
Senior	59 (68.6%)	33 (61.1%)		NS
Senior manager	11 (12.8%)	9 (16.6%)		NS
Emotional exhaustion	10.2 ± 3.5	37.9 ± 6.0	**<0.001**	
Depersonalization	6.6 ± 2.7	17.8 ± 3.9	**<0.001**	
Accomplishment	40.2 ± 2.0	29.6 ± 4.1	**<0.001**	
Job strain				
Job control	64.2 ± 8.4	67.7 ± 9.3	**0.02**	
Job demand	27.1 ± 3.6	27.2 ± 3.5	0.5	
Social support	22.2 ± 4.3	22.1 ± 3.8	**0.8**	
Job satisfaction	7.1 ± 1.5	5.7 ±1.9	**0.01**	
Quality of life (VAS)	7.38 ± 1.29	7.07 ± 1.85	0.25	
HAD anxiety	7.1 ± 2.0	10.1 ± 3.4	**<0.001**	
Score HADa > 10	30 (34.8%)	7 (12.9%)		35.9*, 13.1 (5.5–36.1)
HAD depression	5.5 ± 2.4	6.7 ± 3.4	**0.02**	
Score HADd > 10	0	5 (100%)		

Values are: Mean ± SD or occurrence (%).

* p<0.05.

### Sleep and mental response to chronic work stress in burnout subjects

The group with burnout has significantly higher insomnia troubles, sleep fragmentation, and non-restorative sleep than the control group. In addition, burnout subjects have higher levels of anxiety and depression scores. About job strain characteristics, job control is significantly higher for burnout subjects. In addition, job satisfaction is lower for burnout subjects, and no significant difference was found between the two groups for social support (Tables 1 and 2).

**Table 2:** Sleep parameters in burnout and non-burnout [control] subject.

	Control	Burnout	ANOVA p-value	χ^2^, OR (95% CI)
TST, h	6.9 ± 0.7	6.7 ± 0.8	0.16	
TST week, h	6.7 ± 0.8	6.5 ± 0.9	0.23	
TST week end, h	8.9 ± 1.1	8.8 ± 1.1	0.37	
Difference, h	2.3 ± 1.2	2.3 ± 1.5	0.98	
Epworth Sleepiness Scale (ESS) (/24)	9.7 ± 4.0	9.4 ± 4.8	0.67	
ESS >10, n (%)	46 (53.5%)	28 (51.9%)		NS
ESS > 12, n (%)	26 (30.2%)	17 (31.5%)		NS
ESS > 14, n (%)	16 (18.6%)	11(20.4%)		NS
ESS > 16, n (%)	10 (11.6%)	6 (11.0%)		NS
Insomnia n (%)				
Insomnia	11 (12.8%)	41 (75.9%)		57*, 21.5 (8.8–52.3)
Nb. troubles DSM 5	0.2 ± 0.7	1.6 ± 1.3	**<0.001**	
Sleep latency disorder	3 (3.5%)	11 (29.6%)		19*, 11.6 (3.2–42.4)
Nocturnal awaking	7 (8.1%)	21 (38.9%)		20*, 7.2 (2.8–18.5)
Early awaking	3 (3.5%)	19 (35.2%)		25*,15.1(4.2–54.0)
Non-restorative sleep	7 (8.1%)	27 (50.0%)		32*,11.3 (4.4–28.9)
Nap, n (%)	11 (20.3%)	18 (20.9%)		NS
Snoring n (%)	14 (25.9%)	20 (28.3%)		NS

Values are: Mean ± SD or occurrence (%),

* p<0.05.

Difference = (TST week end—TST week)

### Biological response in burnout subjects

**Fig 1:**
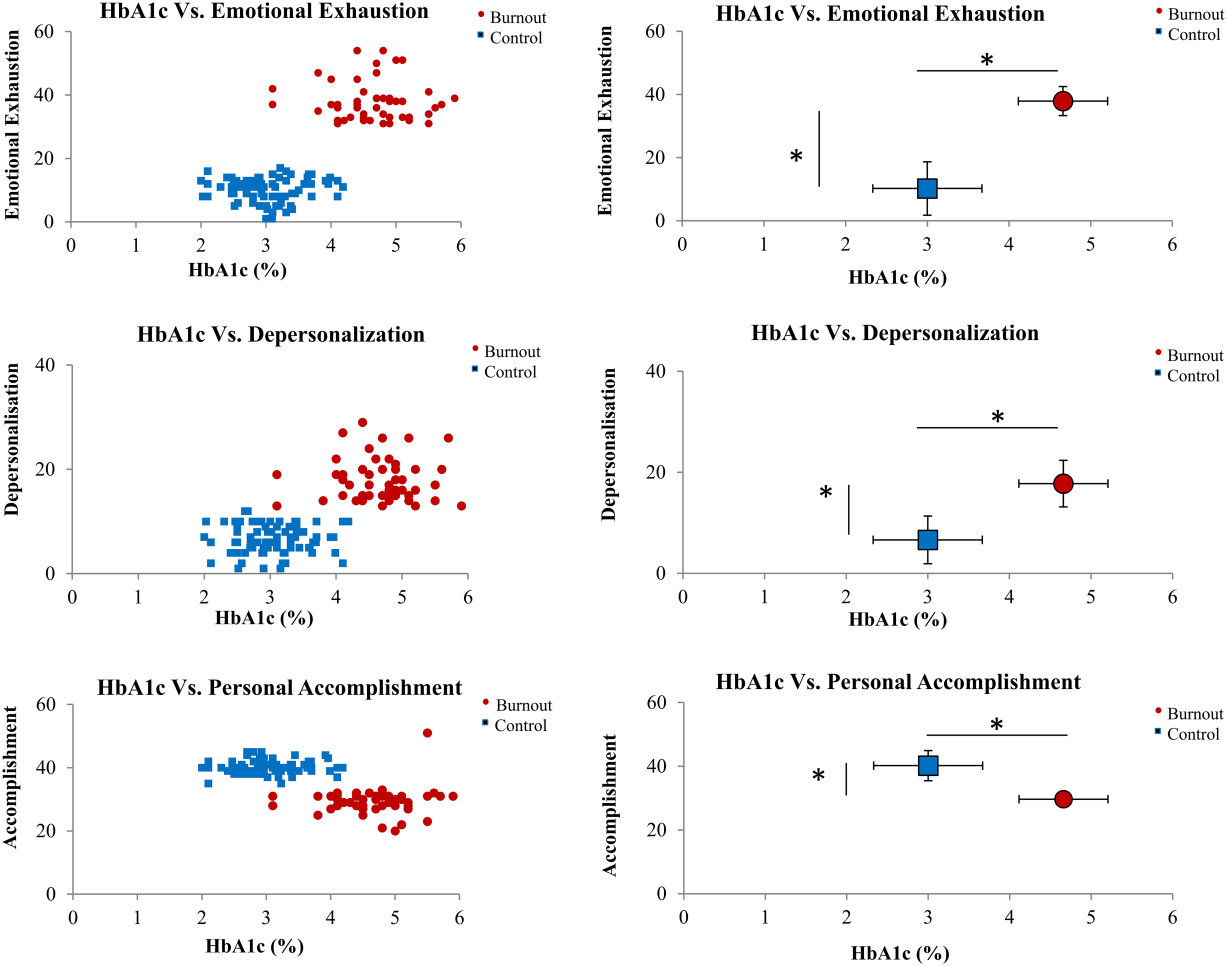
Comparison between HbA1C (individual and mean values (mean ± standard error) and the three burnout dimensions (emotional exhaustion, depersonalization and personal accomplishment) in control and burnout subjects. *p<0.05.

Differences were observed between the two groups of subjects for glycaemia, HbA1C, CRP, 25[OH]D, total-cholesterol, and leukocytes, neutrophils and monocytes cells counts (Table 3). Significant positive correlation (Table 4, Fig 1) existed between HbA1C and two dimensions (EE and DP) of burnout (r = 0.79 and r = 9.71, respectively) and negative correlation between 25[OH]D and EE (r = − 0.85). The third burnout dimension, PA, was negatively correlated with HbA1C (r = − 0.70), glycaemia, CRP and leukocytes and neutrophils counts, and positively with 25[OH]D (r = 0.60).

**Table 3:** Biological parameters in burnout and non-burnout (control) subjects.

	Control	Burnout	p	χ^2^, OR (95% CI)
Glycaemia, g/L	0.82 ± 0.13	0.89 ± 0.14	0.001	
Glycaemia > 0.8 g/L	41 (47.7%)	40 (70.1%)		8.4 *, 1.8 (1.2–2.0)
HbA1C, (%	3.0 ± 0.51	4.66 ± 0.57	<0.001	
HbA1C > 3.5%	14 (16.6%)	52 (60.0%)		78.4*, 4.3 (2.8–6.9)
CRP, mg/L	1.2 ± 0.9	2.07 ± 1.8	0.001	
CRP ≥ 3 mg/L	7 (8.3%)	14 (25.1%)		6.9*, 2 (1.1–3.9)
TSH, mUI/L	1.95 ± 0.63	1.95 ± 0.80	0.999	
25(OH)D, ng/mL	28.9 ± 4.9	17.7 ± 6.9	<0.001	
25(OH)D<20 ng/mL	4 (4.7%)	33 (58.9%)		51.3*, 4.4 (2.9–6.5)
Total-cholesterol, mmol/L	1.66 ± 0.33	1.81 ± 0.38	0.01	
Triglycerides, mmol/L	0.68 ± 0.35	0.74 ± 0.36	0.37	
HDL, mmol/L	0.62 ± 0.16	0.63 ± 0.15	0.48	
LDL, mmol/L	0.98 ± 0.32	1.03 ± 0.34	0.35	
LDL/HDF	0.70 ± 0.28	0.68 ± 0.28	0.78	
Leukocytes, / mm^3^	5110 ± 535	6184 ± 1295	<0.001	
Neutrophils, / mm^3^	2460 ± 420	3418 ± 1248	<0.001	
Eosinophils, / mm^3^	155 ± 99	174 ± 126	0.32	
Basophils, / mm^3^	39.5 ± 17.5	41.1± 19.8	0.61	
Lymphocytes, / mm^3^	2002 ± 488	2068 ± 444	0.42	
Monocytes, / mm^3^	463 ± 115	514 ± 133	<0.001	
Platelets, x 10^3^ / mm^3^	253 ± 49	249 ± 44	0.68	

Values are: Mean ± SD or occurrence (%),

*p<0.05

**Table 4:** The correlation analysis [Pearson coefficient correlation] between the three burnout dimensions [emotional exhaustion, depersonalization and personal accomplishment] and age, job strain [job demand-control-support], job satisfaction, sleep characteristics, anxiety and depression, and biological parameters.

	Emotional Exhaustion	Depersonalization	Personal accomplishment
Age	0.01	0.02	-0.1
Job demand	0.08	0.04	0.02
Job control	0.17*	0.18	-0.12
Job satisfaction	-0.04	-0.31*	0.33*
Social support	-0.05	-0.12	0.09
Nb. sleep troubles (DSM 5)	0.65*	0.45*	-0.51*
ESS	0.01	-0.02	-0.02
TST	-0.1	-0.01	0.09
HADS Anxiety	0.52*	0.45*	-0.50*
HADS Depression	0.26*	0.18	-0.28*
Leucocytes	0.46*	0.44*	-0.44*
PNN	0.45*	0.47*	-0.39*
Glycaemia	0.28*	0.21	-0.26*
HBA1C	0.79*	0.71*	-0.70*
Total-cholesterol	0.22*	0.19*	-0.17
25(OH)D	-0.67*	-0.59*	0.60*
CRP	0.28*	0.15	-0.20*

Values are Pearson coefficient [R]

* is p<0.05

The correlation analysis between biomarkers showed that HbA1C is negatively associated with 25[OH]D (r = − 0.73, p<0.01) and positively with total-cholesterol, glycaemia, CRP and the leukocytes and neutrophils counts (r = 0.56, p<0.01) (Table 5).

**Table 5:** The correlation analysis (Pearson coefficient correlation) between biological parameters.

	**PNN**	**Glycemia**	**HbA1C**	**Cholesterol**	**25(OH)D**	**CRP**
**Leukocytes**	0.86*	0.14	0.54*	0.11	-0.47	0.48
**PNN**		0.14	0.56*	0.17	-0.43	0.56*
**Glycaemia**			0.27*	0.10	-0.30*	0.08
**HbA1C**				0.36*	-0.73*	0.35*
**Cholesterol**					-0.25	0.12
**25(OH)D**						-0.13

Values are Pearson coefficient (R)

* is p<0.05

In the Model 1 multiple logistic regression (Table 6), job control, job satisfaction and anxiety are significantly associated with burnout. These significant associations persist when insomnia is added in the Model 2 (Table 7). However, these models did not significantly predict burnout occurrence (Chi-square = 142 and 131, p = 0.30 and p = 0.50). When HbA1C is included in the Model 1, HbA1C became highly associated with burnout (p<0.001) while job control and job satisfaction were not significantly associated (Model 3, Table 8). When HbA1C and insomnia are included in the Model 1 (Model 4, Table 9), HbA1C remained highly associated with burnout (p<0.001), and insomnia also (p<0.04) (Model 4). In the models 3 and 4, HbA1C significantly predict burnout occurrence (Chi-square = 164 and 166, p = 0.03 for the two). HbA1C has been chosen because its association with burnout was the higher among the biological parameters. Moreover, there are significant correlations between biological parameters associated with burnout.

**Table 6:** Model 1 multiple logistic regression analysis for burnout diagnosis.

Ind. variable	Coefficient	Standard Error	Wald Statistic	p	OR	95%CI
Job satisfaction	-0.46	0.14	9.745	0.002	0.62	(0.41–0.84)
HADS anxiety	0.41	0.11	13.506	<0.001	1.51	(1.21–1.88)
Job control	0.08	0.02	7.657	0.006	1.08	(1.02–1.15)
Job demand	-0.02	0.06	0.143	0.70	0.98	(0.86–1.11)

Pearson Chi-square = 142 (p = 0.30), Likelihood Ratio Test Statistic: 54.0 (P = <0.001)

**Table 7:** Model 2 multiple logistic regression analysis for burnout diagnosis.

Ind. variable	Coefficient	Standard Error	Wald Statistic	p	OR	95%CI
Job satisfaction	-0.41	0.17	5.93	0.02	0.67	(0.48–0.92)
HADS anxiety	0.38	0.13	7.28	0.007	1.40	(1.02–1.79)
Job control	0.08	0.03	6.30	0.01	1.09	(1.02–1.16)
Job demand	-0.05	0.08	0.40	0.53	0.95	(0.82–1.11)
Insomnia	1.26	0.29	19.02	<0.001	3.52	(1.21–6.2)

**Model 2 = model 1 + Insomnia**. Pearson Chi-square = 131 (p = 0.50), Likelihood Ratio Test Statistic: 83.0 (P = <0.001)

**Table 8:** Model 3 multiple logistic regression analysis for burnout diagnosis.

Ind. variable	Coefficient	Standard Error	Wald Statistic	p	OR	95%CI
Job satisfaction	-0.17	0.31	0.31	0.58	0.84	(0.46–1.54)
HADS anxiety	0.42	0.21	4.00	0.05	1.52	(1.01–2.30)
Job control	0.04	0.05	0.62	0.42	1.04	(0.94–1.15)
Job demand	-0.12	0.12	0.89	0.34	0.89	(0.69–1.13)
HbA1C	5.05	1.08	22.0	<0.001	156	(19.2–1293)

**Model 3 = model 1 + HbA1C**. Pearson Chi-square = 164 (**P = 0.03**), Likelihood Ratio Test Statistic: 148.2 (P = <0.001)

**Table 9:** Model 3 multiple logistic regression analysis for burnout diagnosis.

Ind. variable	Coefficient	Standard Error	Wald Statistic	p	OR	95%CI
Job satisfaction	-0.02	0.34	0.03	0.95	0.98	(0.50–1.91)
HADS anxiety	0.34	0.23	2.22	0.13	1.41	(0.89–2.20)
Job control	0.052	0.05	1.06	0.30	1.05	(0.95–1.16)
Job demand	-0.19	0.14	1.84	0.17	0.83	(0.69–1.13)
Insomnia	0.96	0.47	4.17	0.04	0.2.6	(1.11–6.5)
HbA1C	5.42	1.40	14.9	<0.001	226	(14.4–3325)

**Model 4 = model 1 + Insomnia + HbA1C**. Pearson Chi-square = 166 (**P = 0.03**), Likelihood Ratio Test Statistic: 153.9 (P = <0.001)

## Discussion

Our results show that blood concentrations of HbA1C, 25[OH]D and insomnia (particularly the number of troubles) are significantly associated with burnout in our population of white-collar workers. The emotional exhaustion dimension of burnout is particularly associated with the three parameters. In addition, as described previously in the large population (1,300 subjects), insomnia may be a risk factor of burnout, and job control is the only one component of job strain that is associated with the emotional dimension of burnout [32].

A growing base of research has linked disturbances of sleep duration to diabetes, obesity, and cardiovascular disorders, and work stress has been also evidenced to represent a possible risk factor for these diseases [19]. With respect to work stress measurement, job strain, the demand-control-support model of Karasek, is a risk factor of diabetes [33], and high concentration of HbA1C, an index of glucose homeostasis and an indicator of insulin resistance, is associated with high job strain and low social support [18,34]. Work social support is even offered as protective against diabetes diagnosed by HbA1C, while both underload and overload work may increase the risk [35]. To our knowledge, the study of Grossi et al. (2003) [26] evidence that high burnout in women is associated with high levels of HbA1C, independently of confounders including depression. In this study, participants with high scores for burnout reported less control and poorer social support at work, more emotional distress, and greater sleep impairments. According to the literature, we are the first to document that burnout, and particularly emotional exhaustion, the core burnout dimension, is associated with insomnia and HbA1C. We also found higher glycaemia levels in the burnout group but the measurement of HbA1C is recognized for greater monitoring of blood glucose and is more representative of microvascular complications; it has become the gold standard in the assessment of average blood glucose levels over time [36]. At last, subjects with burnout were also significantly higher than controls for the inflammatory biomarker CRP and total-cholesterol, and lower for 25[OH]D concentrations. The leukocyte number and particularly neutrophils and monocytes were also significantly higher. The identification of higher levels of HbA1C and inflammatory biomarkers in burnout together with low levels of 25[OH]D adds information on the latest systematic review which concluded that there are no potential biomarkers for burnout [28]. This review has analyzed 31 burnout studies incorporating 38 different biomarkers and has found any convincing relationship between biomarkers and burnout, due to methodological inconsistencies and vast heterogeneity of this ill-defined syndrome.

It has been evidenced that chronic sleep loss and behavioral or sleep-disorder related represent novel risk factors for weight gain, insulin resistance, and Type 2 diabetes [37]. A recent prospective cohort study demonstrated that long-term excessive daytime napping is associated with the development of elevated HbA1C levels and high HOMA-IR index (homeostasis model assessment of insulin resistance) over an average of 4.5 years of follow-up [38]. Our bivariate analys is showed a significant association between burnout and insomnia, sleep initiation and maintenance disorders or non-restorative sleep. We also found an association with levels of HbA1C when higher than 3.5%, glycaemia (> 0.80 g/L), and CRP (> 3 mg/L). In addition, significant correlations were found between (i) the three dimensions of burnout and HbA1C, 25[OH]D, troubles of insomnia, anxiety and depression, (ii) depersonalization and personal accomplishment and job satisfaction, and (iii) emotional exhaustion and job control. Finally, after adjusting for all potential factors influencing burnout, HbA1c levels and insomnia remain independent statistical risk factors. Our results confirm the Kachi et al [39] study showing that insomnia symptoms (specifically difficulty in maintaining sleep and early morning awakening) have a close association with high HbA1c in a dose-response relationship, in full-time white-collar workers. The association between insomnia and HbA1c level is not clearly elucidated in this study, and authors did not assess the relationship with work stress. Interestingly we confirmed an association between insomnia symptoms and HbA1c levels in the burned-out workers chronically exposed to work stress. In a sense, these two factors could be considered as a bio-clinical signature of chronic stress at workplace. Future prospective research should be conducted to demonstrate the causal pathway. Our findings could also reflect a stress-related dysregulation through multiple neuroendocrine systems. Indeed, Hirotsu et al. [40] has recently underlined the association between sleep disturbance and glucocorticoid dysregulation in response to chronic psychosocial stress. In addition, autonomic and glucocorticoid dysregulations are implicated in metabolic disorders such as the central fat deposition and the metabolic syndrome [41,42].

Another interesting finding of our study is the significant lower level of vitamin D in the burned-out subjects. Vitamin D level seems to be a potent risk factor when lower than 20 ng/mL. In addition, 25[OH]D are in significant negative correlation with HbA1C and glycaemia, and leukocyte count. Recently, low levels of serum 25[OH]D were found associated with short sleep duration and poorer sleep efficiency in a large study of older men [43], and independent inverse associations were found between 25(OH)D and sleepiness (as well as insomnia) [44]. The hypothesis that sleep disorders have become epidemic because of widespread vitamin D deficiency has been proposed, based on the hypothesis of the presence of vitamin D receptors in cerebral areas that play a role in the initiation and maintenance of sleep [45]. On the other hand, available data indicated that both type 1 and type 2 diabetes patients had lower levels of 25[OH]D than controls overall [46]. The mechanism of action of vitamin D in type 2 diabetes is thought to be mediated not only through regulation of plasma calcium levels which regulate insulin synthesis and secretion, but also through a direct action on pancreatic beta-cell function, and its role in inflammation, immunity and gene transcription [47]. In our study, the relationship between burnout and lower levels of 25[OH]D may be inherent to higher HbA1C and glycaemia, that themselves could signed presence of metabolic disorder related to insomnia troubles.

In a broad comprehensive approach encompassing our main findings, we suggest that association between insomnia symptoms, higher HbA1C and lower 25[OH] D levels in a burned-out group could be a specific physiological response to a high level of chronic job strain. In other words, it could also mean that burned employees, all of whom expressed psychological distress at the time of measurement, were close to the "allostatic overload" phase described by McEwen [47]. In our burned-out group, physiological effects could be delayed compared to the psychological effects of burnout. Moreover, insomnia symptoms might be either a protective mechanism for maintaining physical health or a step before irreversible damaging effects of stress. It has been suggested in some other studies cited by Melamed et al. (2006) [27] that allostatic load could represent a possible mechanism explaining the physical health consequences of burnout. A high allostatic load disrupts dynamic biological responses, resulting notably in inadequate biological responses, and higher risk for chronic diseases such as hypertension, obesity, cardiovascular disease, and diabetes [47,48]. The chronic allostatic load can be a mechanism by which stress exposure contributes to the risk of diabetes and is also implicated in the adverse health consequences of diabetes [34].

This study has several limitations, and caution should be taken when interpreting the results. First, this study was a descriptive, cross-sectional research design, therefore it was not possible to analyze the causal relationships between the variables. A prospective study is required to examine the link between burnout and HbA1C and 25[OH]D serum levels in our population of white-collar workers [19]. Secondly, the use of self-reporting questionnaires may have influenced the sample and the results. For example, burned-out subjects may have respond more negatively because of their negative felling at the time they filled out the questionnaire. In the future, it would be interesting for example to objectively investigate inter-relationship between elevated levels of HbA1C and sleep characteristics (e.g., fragmentation) using ultra-miniaturized PSG as previously used by us [49]. Moreover, we did not record the length of burnout experience that could be pertinent to study the causal link with the development of chronic inflammatory or metabolic diseases and/or the sleep disorders.

In conclusion, this study evidenced that serum levels of HbA1C higher than 3.5% and 25[OH]D lower than 20 ng/mL and insomnia symptoms are closely associated with burnout in white collar workers. Biological parameters and sleep disorders should be an interesting path to explore health consequences of burnout at work place. Thus, for better characterization of work-related burnout it would be interesting to develop an allostatic approach including subjective and objective sleep parameters that could open up new perspectives for research. Future studies should be prospective and focused on high job strain workers such as physicians or the large group of medical occupations who are more prone to burnout prevalence. At the end, our results confirm the need for enhancing the primary prevention in the workplace through social-behavioral approaches and management techniques.
